# Algorithm for screening and management of locomotive syndrome in elderly individuals and development of a short version of the 25-question Geriatric Locomotive Function Scale-Portuguese

**DOI:** 10.31744/einstein_journal/2022AO6349

**Published:** 2022-11-18

**Authors:** Jessica Anelise Parreira Alves, Daniela Regina Brandao Tavares, Jane Erika Frazao Okazaki, Maria Carolyna Fonseca Batista Arbex, Júlia de Carvalho Galiano, Sabrina Nascimento do Carmo, Fânia Cristina dos Santos

**Affiliations:** 1 Universidade Federal de São Paulo São Paulo SP Brazil Universidade Federal de São Paulo, São Paulo, SP, Brazil.

**Keywords:** Aged, Locomotion, Surveys and questionnaires, Validation study, Algorithms

## Abstract

**Objective:**

To develop a short version of the 25-question Geriatric Locomotive Function Scale-Portuguese and to create an algorithm for locomotive syndrome screening and management.

**Methods:**

The 25-question Geriatric Locomotive Function Scale-Portuguese was applied to individuals aged 60 years or older seen at the Geriatrics and Gerontology Department of *Universidade Federal de São Paulo* , between 2016 and 2018. Items of the 25-question Geriatric Locomotive Function Scale-Portuguese were submitted to exploratory factor analysis using the principal component method. Internal consistency was investigated using Cronbach’s alpha coefficient. The ROC curve was used to determine the cut-off point of the short version developed. Finally, a simple and objective algorithm was created for locomotive syndrome screening and management using the Delphi method.

**Results:**

A total of 202 elderly individuals aged 61 to 101 years (mean age, 84.67 years) were evaluated. Fifteen items were excluded from the 25-question Geriatric Locomotive Function Scale-Portuguese to compose the 10-question Geriatric Locomotive Function Scale-Portuguese, a 10-item instrument with appropriate psychometric properties. A cut-off point of ten (ROC curve) was determined for potential locomotive syndrome, with 96.5% sensitivity and 86.2% specificity. A very simple algorithm was developed for locomotive syndrome screening and management.

**Conclusion:**

The short version (10-question) of the Geriatric Locomotive Function Scale-Portuguese has appropriate psychometric properties and provides a practical tool for detection of locomotive problems in elderly individuals.

## INTRODUCTION

For the last two decades, the World Health Organization (WHO) has been emphasizing the socioeconomic impact of population aging and the need for health promotion strategies aimed at the elderly population.^( 1 , 2 )^ The rising number of older adults affects family structures, public policy demands and resource distribution in the society.^( 3 )^ In Brazil, the elderly population is expected to increase by 239.0% (from 19.6 million in 2010 to 66.6 million between 2010 and 2050) according to Brazilian Institute of Geography and Statistics (IBGE - *Instituto Brasileiro de Geografia e Estatística* ) estimates.^( 3 )^

Locomotion ability is one of the most important determinants of healthy aging.^( 1 )^ Musculoskeletal and joint diseases rank second as major cause of years lived with disability, following cardiovascular diseases.^( 4 )^ However, current health strategies addressing musculoskeletal system disorders in elderly individuals in Brazil are disappointing.

The musculoskeletal system consists of muscles, joints, cartilages, bones, intervertebral discs and nerves. Muscles and bones naturally become weaker with age. However, sedentary lifestyle plays a major role in this process and may progress to sarcopenia and osteoporosis.^( 5 )^ Pain and movement restriction due to osteoarthritis may enhance muscle and bone weakening, which, in turn, leads to further joint degeneration. Hence, the musculoskeletal system structures undergo a slow, prolonged and interrelated deterioration process, eventually leading to loss of functionality.^( 6 )^

The Katz index and Lawton scale are widely used to assess functional independence in basic and instrumental activities of daily living, respectively.^( 7 , 8 )^ However, since functional decline may involve other causes, such as cognitive impairment, these instruments are not specific for loss of functionality due to musculoskeletal dysfunction.^( 8 )^ Also, these instruments are not amenable to early detection of loss of independence, which is the primary goal.

In 2007, the Japanese Orthopaedic Association (JOA) proposed the term “locomotive syndrome” (LS) as part of a health prevention strategy.^( 6 , 9 )^ “Locomotive syndrome” is an epidemiological concept with impact on health care system management. This syndrome is defined as a condition with high risk of inability to walk and dependence in activities of daily living due to locomotive dysfunction.^( 5 , 10 )^ The 25-Question Geriatric Locomotive Function Scale was developed for LS diagnosis.^( 6 )^ A translated, cross-culturally adapted and validated version of this scale (25-question Geriatric Locomotive Function Scale-Portuguese, GLFS 25-P is already available in Brazil.^( 11 )^

However, the GLFS 25-P is a long and nonpractical scale. Given several tests and questionnaires are often applied during consultations in public geriatric care settings, simplified assessment instruments may offer a more practical and less time consuming alternative, with better acceptance by patients.^( 12 )^ Short versions of assessment instruments also enable collection of data from large population groups.^( 12 )^

## OBJECTIVE

To develop a short version of the 25-question Geriatric Locomotive Function Scale-Portuguese and to create an algorithm for locomotive syndrome screening and management.

## METHODS

A methodological, descriptive, and analytical study approved by the Ethics Committee of *Universidade Federal de São Paulo* (UNIFESP), CAAE: 37238614.7.0000.5505; # 921.390. All participants signed an Informed Consent Form.

Male and female elderly individuals aged 60 years or older and seen at the outpatient unit of UNIFESP Geriatrics and Gerontology Department, between 2016 and 2018, were selected. The following exclusion criteria were applied: severe acute or chronic decompensated disease, limiting sensory *deficit* , fracture of any kind in the last six months or cognitive impairment (defined by Mini-Mental State Examination scores lower than the literacy cut-off, as follows: illiteracy, 20 points; 1 to 4 years of education, 25 points; 5 to 8 years of education, 26 points; 9 to 11 years of education, 28 points; more than 11 years of education, 29 points).^( 13 )^

Semi-structured questionnaires and some instruments were individually administered in outpatient facilities. Data collection and assessment instrument administration were carried out by trained investigators. Training included several aspects of the study for homogenization purposes. Investigators were not professionally related to participants.

Sociodemographic aspects were addressed and functional status regarding basic and instrumental activities of daily living determined according to the Katz index and Lawton scale, respectively. Katz index scores range from zero to six (six, total dependency; zero, independency).^( 7 , 14 )^ In the Lawton scale, dependency levels are categorized as follows: 9, totally dependent; 10 to 15, severely dependent; 16 to 20, moderately dependent; 21 to 25, mildly dependent; 26 to 27, independent.^( 8 , 15 , 16 )^

The GLFS 25-P was also administered. This instrument comprises 25 questions. Responses are rated zero to four and the final score ranges from zero to 100. The higher the score, the greater the locomotive impairment. In the validation study in the Brazilian population, LS diagnosis was defined as total score equal to or greater than 19.^( 11 )^

### Development of the short version of the 25-question Geriatric Locomotive Function Scale-Portuguese

The dimensionality of the GLFS 25-P was assessed using factor analysis. Factors with eigenvalues higher than 1 were selected. Items with commonalities and factor loadings lower than 0.7 were excluded. Kaiser-Meyer-Olkin sample adequacy coefficients and the Bartlett test of sphericity were used to assess the overall significance of correlations between scale items. The Cronbach’s alpha coefficient was calculated for analysis of items composing each factor. A ROC curve was constructed to determine the cut-off score of the short version of the GLFS 25-P. The cut-off score 19 of the long version of GLFS 25-P validated in Brazil was used as a reference.^( 11 )^

### Statistical analysis

Data were analyzed using the (SSPS), version 2. Mean, minimum and maximum values for age and the frequency of variables were calculated for descriptive analysis. Principal components analysis, Varimax orthogonal rotation, Kaiser-Meyer-Olkin coefficients and the Bartlett test of sphericity were used in factor analysis. For inferential analysis of data, the internal consistency was measured using Cronbach’s alpha. The level of significance was set at 5% (p≤0.05).

### Development of an algorithm for locomotive syndrome screening and management

Algorithms consist of a finite sequence of well-defined instructions carried out in a systematic way. These tools are particularly useful to obtain a broad view in process organization. In this study, a panel of eight specialists was invited to participate in subsidiary steps of development of the LS screening and management algorithm. This panel included professionals specialized in locomotion, geriatrics, and LS, with 3.5 to 28 years of experience.

First, a narrative review of the scientific literature was carried out by one of the panel specialists. The Latin American and Caribbean Literature on Health Sciences (LILACS) and MEDLINE^®^ databases were searched. Narrative reviews are the most comprehensive and appropriate for description and discussion of the development or state of the art of a given topic, from the theoretical or the contextual perspective. Articles published in Portuguese or English in the last 12 years and containing the keyword “locomotive syndrome” were selected.

Abstracts were simultaneously selected and read by a panel of eight specialists. Studies addressing LS screening, assessment and management were selected. The Delphi method was used to reach a group consensus about the topic of interest ( *i.e.* , consensus building based on a systematic approach, with active and collective participation of all specialists in data relevance assessment).^( 17 )^ This process was completed in two meetings lasting approximately two hours each.

Topics relevant for the creation of a simple and practical LS assessment algorithm were discussed, such as best screening and diagnostic methods for musculoskeletal problems.

## RESULTS

The sample comprised 202 elderly individuals aged 61 to 101 years (mean age, 84.6 years). The following characteristics prevailed: female sex (73%), white skin color (55%), widower marital status (57%), low level of education (1 to 4 years of education, 55.5%) and functional independency (total independency in basic and instrumental activities of daily living, 63.3% and 48%, respectively) ( Table 1 ).

**Table 1:** Sample characterization

Characteristics		
Age, years		
	Mean (Min.-Max.)	84.6 (61-101)
	60-70	9 (4.5)
	71-80	30 (14.8)
	81-90	129 (63.9)
	>90	34 (16.8)
Sex		
	Male	54 (27)
	Female	148 (73)
Color		
	White	112 (55)
	Brown	58 (29)
	Yellow	19 (6)
	Black	12 (9.5)
	Indigenous	1 (0.5)
Marital status		
	Widower	116 (57)
	Married	64 (32)
	Single	12 (6)
	Divorced	10 (5)
Level of education, years		
	Illiterate	30 (15)
	1-4	112 (55.5)
	5-8	33 (16)
	9-11	9 (4.5)
	≥12	18 (9)
BADL		
	Independent	128 (63.3)
	Dependent for 1 activity	68 (33.7)
	Dependent for 2 activities	4 (2)
	Dependent for 3 activities	1 (0.5)
	Dependent for 4 activities	0
	Dependent for 5 activities	0
	Dependent for 6 activities	1 (0.5)
IADL		
	Independent	97 (48)
	Mildly dependent	79 (39)
	Moderately dependent	19 (9.5)
	Severe dependent	7 (3.5)

Except when stated otherwise, results are expressed in n (%).

BADL: basic activities of daily living; IADL: instrumental activities of daily living; Min.-Max.: Minimum-Maximum.

Initial GLFS 25-P scale dimensionality assessment (exploratory factor analysis) revealed five factors, each comprising the following questions: factor 1, questions 5, 6, 7, 8, 9, 10, 11, 14, 17 and 19; factor 2, questions 12, 13, 15, 18, 20 and 21; factor 3, questions 16, 22 and 23; factor 4, questions 2, 3 and 4; factor 5, questions 1, 24 and 25. These five factors explained 67.2% of total item variance.

Items with commonality lower than 0.7 and factor loadings lower than 0.7 were used as exclusion criteria. Items with variance due to common factors less than 70% ( *i.e.* , poorly represented in factor analysis) were excluded. Items were removed one by one and further factor analyses run after each removal. Fourteen steps were required for removal of all items with commonality lower than 0.7 ( Table 2 ).

**Table 2:** Exploratory factor analysis of items removed and respective commonalities

Step	Item removed	Commonality
1	Q17. To what extent has it been difficult to carry objects weighing 2kg?	0.53
2	Q19. To what extent have simple tasks and housework been difficult?	0.55
3	Q14. To what extent has it been difficult to keep yourself neat?	0.58
4	Q1 Did you have any pain in your neck or upper limbs?	0.59
5	Q8. To what extent has it been difficult to put on and take off shirts?	0.62
6	Q2 Did you have any pain in your back, lower back, or buttocks?	0.47
7	Q4. To what extent has it been painful to move your body in daily life?	0.50
8	Q3 Did you have any pain in your lower limbs?	0.55
9	Q9. To what extent has it been difficult to put on and take off trousers and pants?	0.59
10	Q11. To what extent has it been difficult to wash your body in the bath?	0.66
11	Q16. To what extent has it been difficult to go out to visit neighbors?	0.68
12	Q22. Have you been restricted from meeting your friends?	0.52
13	Q23. Have you been restricted from joining social activities?	0.45
14	Q12. To what extent has it been difficult to go up and down stairs?	0.68

Q: question.

Three factors were extracted in factor analysis run after removal of 14 items: factor 1, comprising questions 13, 15, 18, 20 and 21, factor 2, comprising questions 5, 6, 7 and 10; and factor 3, comprising questions 24 and 25. Item 18 was removed due factor loading lower than 0.7 ( Table 3 ). Hence, the three remaining factors explained 74.9% of total variance of data. The selection of this number of factors was based on the number of eigenvalues higher than 1 in the correlation matrix ( Table 4 ).

**Table 3:** Factor loading analysis

Questions	Factor loading		
	Factor 1	Factor 2	Factor 3
Q21. To what extent has it been difficult to perform sports activities?	0.843	0.199	0.126
Q13. To what extent has it been difficult to walk briskly?	0.831	0.357	0.044
Q15. How far can you keep walking without rest?	0.794	0.247	0.141
Q20. To what extent have load-bearing tasks and housework been difficult?	0.769	0.234	0.301
Q18. To what extent has it been difficult to use public transportation?	0.682	0.441	0.216
Q10. To what extent has it been difficult to use the toilet?	0.167	0.849	0.135
Q5. To what extent has it been difficult to get up from a bed or lie down?	0.235	0.804	0.111
Q7. To what extent has it been difficult to walk inside the house?	0.266	0.782	0.183
Q6. To what extent has it been difficult to stand up from a chair?	0.403	0.747	0.114
Q25. Have you ever felt anxious about being unable to walk in the future?	0.170	0.067	0.870
Q24. Have you ever felt anxious about falls in your house?	0.148	0.255	0.808

Q: question.

**Table 4:** Eigenvalues, percentage of explained variance and Cronbach’s alpha coefficient of the three scale factors

	Factor 1	Factor 2	Factor 3
Eigenvalues	5.10	1.22	1.17
Percentage (%) of total variance explained	29.91	28.87	16.16
Cronbach’s alpha	0.878	0.868	0.687

**Figure 1:**
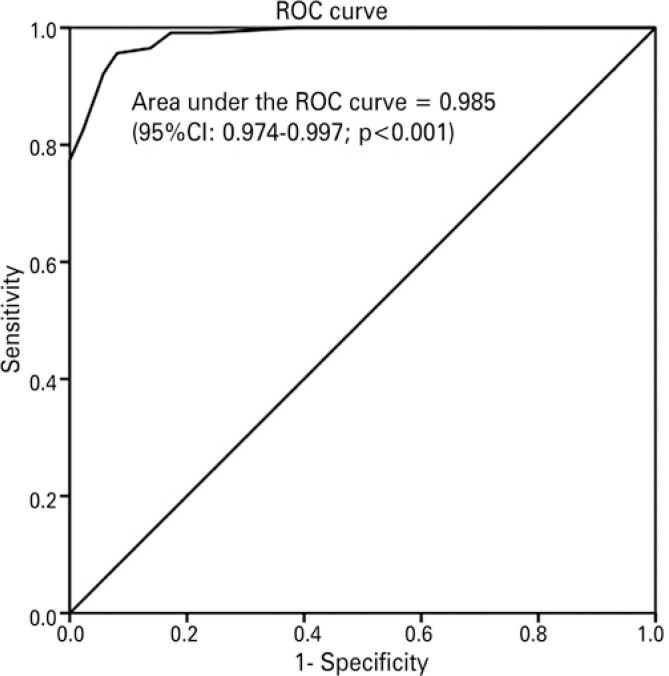
ROC curve of the 10-question Geriatric Locomotive Function Scale

The internal consistency of retained factors varied between 0.88 and 0.69 (Cronbach’s alpha coefficient). Hence, a reliable short version of the GLFS 25-P was obtained. This version was named 10-question Geriatric Locomotive Function Scale (GLFS 10-P) (Appendix 1). The ROC curve constructed for the GLFS 10-P indicated a cut-off score of 10, with 96.5% sensitivity and 86.2% specificity for LS diagnosis ( Figure 1 ).

The older the participant, the higher the scores obtained in the GLFS 10-P (correlation analysis of age and LS) ( Table 5 ). The sensitivity and specificity of the GLFS 10-P did not differ between non-long-living (60 to 79 years) and long-living individuals (80 years or older) ( Table 6 ).

**Table 5:** Correlations between the 10-question Geriatric Locomotive Function Scale and age

GLFS 10-P	Age (years)				p value *
	60-70 (n=9)	71-80 (n=30)	81-90 (n=131)	≥91 (n=32)	
<10	5/9 (55.6)	20/30 (66.7)	50/131 (38.2)	4/32 (12.5)	<0.001
≥10	4/9 (44.4)	10/30 (33.3)	81/131 (61.8)	28/32 (87.5)	

* descriptive power of the χ^2^ test.

GLFS 10-P: 10-question Geriatric Locomotive Function Scale.

**Table 6:** Sensitivity and specificity of the 10-question Geriatric Locomotive Function Scale for long-living and non-long-living elderly individuals

GLFS 10-P score	Total	Age (years)		p value*
		60-79	≥80	
Sensitivity	96.5 (91.3-99.0)	100.0 (75.3-100.0)	96.1 (90.3-98.9)	1.000
Specificity	86.2 (77.1-92.7)	95.2 (76.2-99.9)	83.3 (72.1-91.4)	0.279

Results expressed as 95% confidence intervals.

* descriptive power of the χ^2^ test.

GLFS 10-P: 10-question Geriatric Locomotive Function Scale.

### Algorithm

Of note, 97 articles were initially selected for algorithm development. Of these, 20 were used in the narrative review. A minimum interrater agreement of 80% was required for algorithm development.

Instruments and tests provide a broad view of approaches. Therefore, a map comprising relevant elements for LS assessment was created. The first step consisted of search of suspected cases. The following steps involved assessment of cases for probable diagnosis of LS, confirmed diagnosis of LS and determination of LS severity.

**Figure 2:**
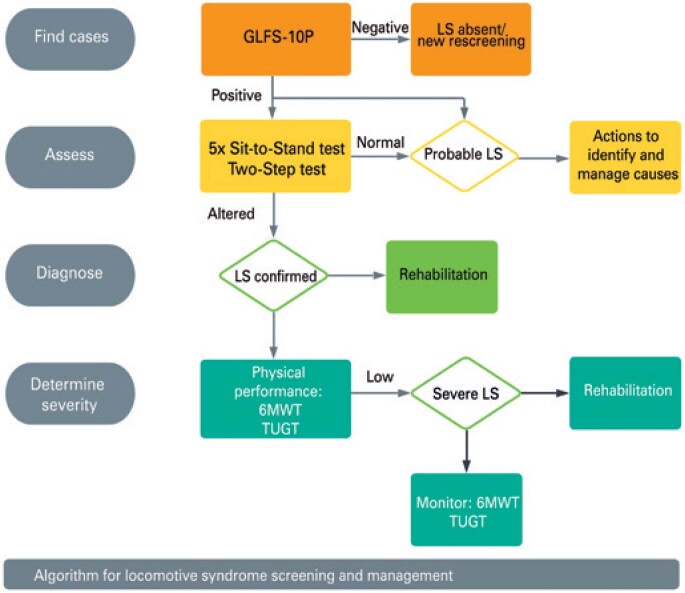
Algorithm for locomotive syndrome screening and management

The final algorithm proposed for LS screening and management in elderly patients included four steps ( Figure 2 ). In the first step (“LS screening”), the GLFS 10-P was used to identify suspected cases. Probable LS was defined as scores ≥10. In the next step (“assessment”), application of the 5x Sit-to-Stand test and the Two-Step test were introduced. Normal test results suggested locomotive impairment risks associated with musculoskeletal structures, *as per* the GLFS 10-P. In these cases, actions aimed at diagnosis and early treatment of underlying causes of potential LS are indicated. The following time cut-offs were adopted in the 5x Sit-to-Stand test: up to 11.4, 12.6 and 14.8 seconds (60 to 69 years, 70 to 79 years and 80 years or older age ranges, respectively).^( 18 )^ In the Two-Step test, values equal to or higher than 1.3cm/cm were defined as normal.^( 19 )^

GLFS 10-P: 10-question Geriatric Locomotive Function Scale; LS: locomotive syndrome; 6MWT: 6-minute walk test; TUGT: Timed Up and Go Test.

The third step (diagnosis) consisted of overt LS identification ( *i.e.* , confirmed diagnosis of LS according to physical performance changes (5x Sit-to-Stand or Two-Step test) and specific rehabilitation prescription. The fourth step (determination of LS severity) was based on Timed Up and Go and 6-minute walk test results. Changes in any of these tests suggested severe LS and need of continuous rehabilitation and monitoring.^( 20 , 21 )^

## DISCUSSION

This is the first study to propose a short version of the GLFS 25-P for fast and pratical secreening of musculoskeletal problems in elderly individuals. Availability of such simple tools may assist professionals in services with large demand.^( 22 )^

This study comprised primarily female participants (73%), supporting the phenomenon of feminization of aging, described in the scientific literature.^( 23 )^ Large numbers of participants with advanced age (80.7% aged >80 years) in this sample are also representative of the fastest growing age group worldwide ( *i.e.* , long-living individuals).^( 23 , 24 )^ Despite wide age variation, sensitivity and specificity of the GLFS 10-P did not differ between non-long-living (60 to 79 years) and long-living (80 years or older) elderly individuals in this study.

According to the GLFS 10-P, the prevalence of probable LS among elderly individuals in this sample was 61%. This finding suggests a high proportion of elderly individuals are at risk of losing functional independency due to locomotive dysfunction.^( 5 )^

The short version of the GLFS 25-P (GLFS 10-P) is a 10-question instrument with appropriate internal consistency (between 0.88, 0.87 and 0.69), as per the Cronbach’s alpha coefficient. Values equal to or greater than 0.70 are deemed acceptable.^( 19 )^ Psychometric measures did not differ between the short (GLFS 10-P) and the long (GLFS 25-P) versions.^( 10 )^

In the original scale, several questions are very similar - questions addressing social interaction (items 16, 22 and 23), for example. In the short version developed here was able to reduce some of these redundancies without, however, invalidating the measurement properties. Creation of a shorter version of the GLFS 25-P without detriment to psychometric characteristics may encourage wider use of this instrument.

This study set out to develop a simple algorithm for early detection of locomotion-related functional impairment risks in elderly individuals. Instruments such as the Katz index and Lawton scales are often used for functional assessment of elderly individuals. However, these instruments provide a delayed assessment of functional decline relative to the GLFS 25-P. In a study with functional assessment of elderly individuals for LS according to the GLFS-25 and performance in instrumental activities of daily living, Tobimatsu reported the GLFS-25 enabled earlier detection of functional decline in this age group.^( 25 )^ In another study, Arbex et al. observed LS was significantly correlated with functionality in daily living (basic and instrumental activities), non-chronic pain (OR: 15.92; 95% confidence interval – 95%CI: 3.08-82.27) and worse self-perception of health (OR: 0.23; 95%CI: 0.07-0.79), which are important factors for the quality of life of the elderly.^( 26 )^

The GLFS 10-P proved to be a valid and practical screening tool for detection of suspected cases of LS. Therefore, application of this instrument is recommended in the first step of the algorithm proposed. In the next steps, application of the 5x Sit-to-Stand test: or the Two-Step test and the gait speed or the Timed Up and Go test are indicated to complement the diagnostic process and determine LS severity, respectively. Processes described in this study are particularly useful for engagement of elderly individuals in rehabilitation.^( 26 )^ Correlations of several tests with the GLFS-25, including the Timed Up and Go and the gait speed tests, have been reported by Muramoto et al. However, multivariate analysis in that study revealed stronger correlations with the Timed Up and Go than with the gait speed test. This may have reflected the higher demands of the Timed Up and Go test ( *i.e.* , more complex movements, such as getting up, turning, stopping, and sitting, rather than just walking on the horizontal plane).^( 27 )^

As to limitations of this study, questionnaires and scales, such as the GLFS 25-P and the GLFS 10-P, rely on subjective perceptions of individuals evaluated.^( 27 )^ This is precisely why combination with physical performance tests ( *i.e.* , more objective tests) is recommended. Development of the short version of the GLFS 25-P was based on a small sample obtained from a single organization. On the other hand, the sample comprised primarily long-living elderly individuals (mean age of 84 years). This age group is still poorly investigated, in spite of being the fastest growing population group.^( 23 , 24 )^ Also important, the literature review carried out for algorithm development was limited to the LILACS and MEDLINE^®^ databases and only articles published in Portuguese or English were included.

Application of the short version GLFS 10-P to population samples from other Brazilian regions is warranted for more accurate analysis of validity parameters. Likewise, the algorithm proposed should be used in other regions of the country to determine its true utility.

## CONCLUSION

A short version of the 25-question Geriatric Locomotive Function Scale-Portuguese comprising only 10 questions was developed. The 10-question Geriatric Locomotive Function Scale is a simple instrument with appropriate measurement properties, which may contribute to wider use in clinical practice. The algorithm proposed is aimed to facilitate assessment of locomotive syndrome and is a promising tool for management of functional disability secondary to musculoskeletal problems.

## Appendix 1.

Short version of the 25-question Geriatric Locomotive Function Scale-Portuguese (GLFS 25-P) supplementary material


