# Supplementary material for: Algorithm for screening and management of locomotive syndrome in elderly individuals and development of a short version of the 25-question Geriatric Locomotive Function Scale-Portuguese
Source: Einstein (Sao Paulo). 2022 Nov 18;20:eAO6349. doi: 10.31744/einstein_journal/2022AO6349 (PMC9744431; doi:10.31744/einstein_journal/2022AO6349)
Supplement: Supplementary file 1 [file 2317-6385-eins-20-eAO6349-app1.pdf]

Appendix 1. Short version of the 25-question Geriatric Locomotive Function Scale-Portuguese (GLFS 25-P) supplementary material

ESCALA GERIÁTRICA DA FUNÇÃO LOCOMOTORA DE 10 ITENS – GLFS 10-P *As perguntas a seguir se referem à sua condição de saúde e atividades de vida diária, relacionados a suas costas, membros inferiores e superiores. Por favor, responda considerando sua condição no último mês:*					
	0 ponto	***1 ponto***	***2 pontos***	***3 pontos***	***4 pontos***
1) Até que ponto tem sido difícil praticar atividades esportivas?	*Sem dificuldade*	*Pouca dificuldade*	*Moderada dificuldade*	*Muita dificuldade*	*Extrema dificuldade*
2) Até que ponto tem sido difícil andar rápido?	*Sem dificuldade*	*Pouca dificuldade*	*Moderada dificuldade*	*Muita dificuldade*	*Extrema dificuldade*
3) Quanto você consegue andar sem descansar?	*Mais de 2-3Km*	*Aproximadamente 1Km*	*Aproximadamente 300m*	*Aproximadamente 100m*	*Aproximadamente 10m*
4) Até que ponto tem sido difícil realizar as tarefas pesadas do lar?	*Sem dificuldade*	*Pouca dificuldade*	*Moderada dificuldade*	*Muita dificuldade*	*Extrema dificuldade*
5) Até que ponto tem sido difícil usar o banheiro?	*Sem dificuldade*	*Pouca dificuldade*	*Moderada dificuldade*	*Muita dificuldade*	*Extrema dificuldade*
6) Até que ponto tem sido difícil levantar da cama ou deitar?	*Sem dificuldade*	*Pouca dificuldade*	*Moderada dificuldade*	*Muita dificuldade*	*Extrema dificuldade*
7) Até que ponto tem sido difícil caminhar dentro de casa?	*Sem dificuldade*	*Pouca dificuldade*	*Moderada dificuldade*	*Muita dificuldade*	*Extrema dificuldade*
8) Até que ponto tem sido difícil levantar da cadeira?	*Sem dificuldade*	*Pouca dificuldade*	*Moderada dificuldade*	*Muita dificuldade*	*Extrema dificuldade*
9) Você já se sentiu com medo de não poder andar no futuro?	*Nunca*	*Quase nunca*	*Às vezes*	*Quase sempre*	*Sempre*
10) Você já se sentiu com medo de cair dentro de casa?	*Nunca*	*Quase nunca*	*Às vezes*	*Quase sempre*	*Sempre*
